# Publisher Correction: Pla2g12b drives expansion of triglyceride-rich lipoproteins

**DOI:** 10.1038/s41467-024-46847-y

**Published:** 2024-03-22

**Authors:** James H. Thierer, Ombretta Foresti, Pradeep Kumar Yadav, Meredith H. Wilson, Tabea O. C. Moll, Meng-Chieh Shen, Elisabeth M. Busch-Nentwich, Margaret Morash, Karen L. Mohlke, John F. Rawls, Vivek Malhotra, M. Mahmood Hussain, Steven A. Farber

**Affiliations:** 1 Department of Embryology, Carnegie Institution for Science, Baltimore, MD 21218 USA; 2 Johns Hopkins University in Baltimore, Maryland Department of biology, Baltimore, MD 21218 USA; 3 Centre for Genomic Regulation (CRG), The Barcelona Institute for Science and Technology, Barcelona, 08003 ES Spain; 4 Department of Foundations of Medicine, NYU Long Island School of Medicine, Mineola, NY 11501 USA; 5 Department of Botany, Faculty of Science, University of Allahabad, Prayagraj, India; 6 School of Biological and Behavioural Sciences, Queen Mary University of London, London, E1 4NS GB UK; 7 Department of Molecular Genetics and Microbiology, Duke University, Durham, NC 27708 USA; 8 Department of Genetics, University of North Carolina, Chapel Hill, NC 27599 USA; 9 Universitat Pompeu Fabra, Barcelona, Spain; 10 Institució Catalana de Recerca i Estudis Avançats, Barcelona, Spain

**Keywords:** Genetics, Metabolic disorders, Lipoproteins

Correction to: *Nature Communications* 10.1038/s41467-024-46102-4, published online 07 March 2024

In this article the affiliation details for Steven A. Farber were incorrectly given as ‘Department of Botany, Faculty of Science, University of Allahabad, Prayagraj, India.’ but should have been 'Department of Biology, Johns Hopkins University, Baltimore, MD 21218, USA’.

In addition, the affiliation details for Vivek Malhotra were incorrectly given as ‘Department of Biology, Johns Hopkins University, Baltimore, MD 21218, USA.’ but should have been ‘Centre for Genomic Regulation (CRG), The Barcelona Institute for Science and Technology, Barcelona 08003 ES, Spain’. The following affiliations were also omitted and have now been included: ‘Universitat Pompeu Fabra, Barcelona, Spain.’ and ‘Institució Catalana de Recerca i Estudis Avançats, Barcelona, Spain.’

In addition, the affiliation details for M. Mahmood Hussain were incorrectly given as ‘Centre for Genomic Regulation (CRG), The Barcelona Institute for Science and Technology, Barcelona 08003 ES, Spain’ and should have been ‘Department of Foundations of Medicine, NYU Long Island School of Medicine, Mineola, NY 11501, USA.’

The original article has been corrected.

