# Supplementary material for: Pla2g12b drives expansion of triglyceride-rich lipoproteins
Source: Nat Commun. 2024 Mar 7;15:2095. doi: 10.1038/s41467-024-46102-4 (PMC10920679; doi:10.1038/s41467-024-46102-4)
Supplement: Supplementary file 1 — Supplementary Information [file 41467_2024_46102_MOESM1_ESM.pdf]

Supplementary Information

Pla2g12b Drives Expansion of Triglyceride-Rich Lipoproteins

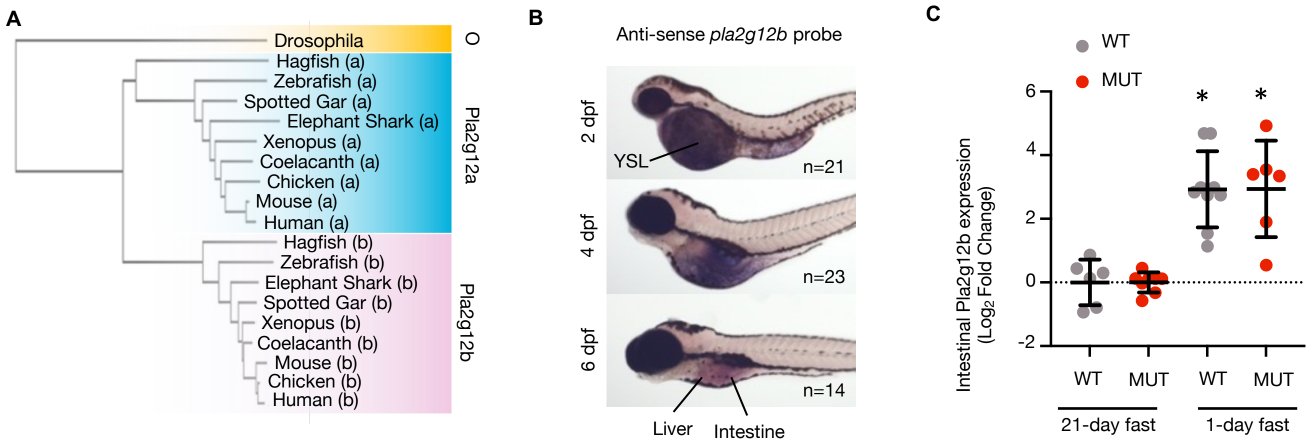

Supplementary Fig. 1: *pla2g12b* expression correlates with TRL production. (A) Phylogenetic tree of *Pla2g12b* and *Pla2g12a*, showing both ohnologs are universally conserved throughout the vertebrate lineage. The invertebrate phospholipase A2 from *Drosophila melanogaster* is shown as an outgroup. **(B)** Whole-mount *in situ* hybridization of *pla2g12b* expression throughout larval zebrafish development demonstrating strong expression in lipoprotein-producing tissues (YSL, liver, and intestine). **(C)** qPCR of *pla2g12b* gene expression in adult zebrafish intestines following 1 or 21-day fasting periods. Welch's t-test, an asterisk denotes $p < 0.005$.

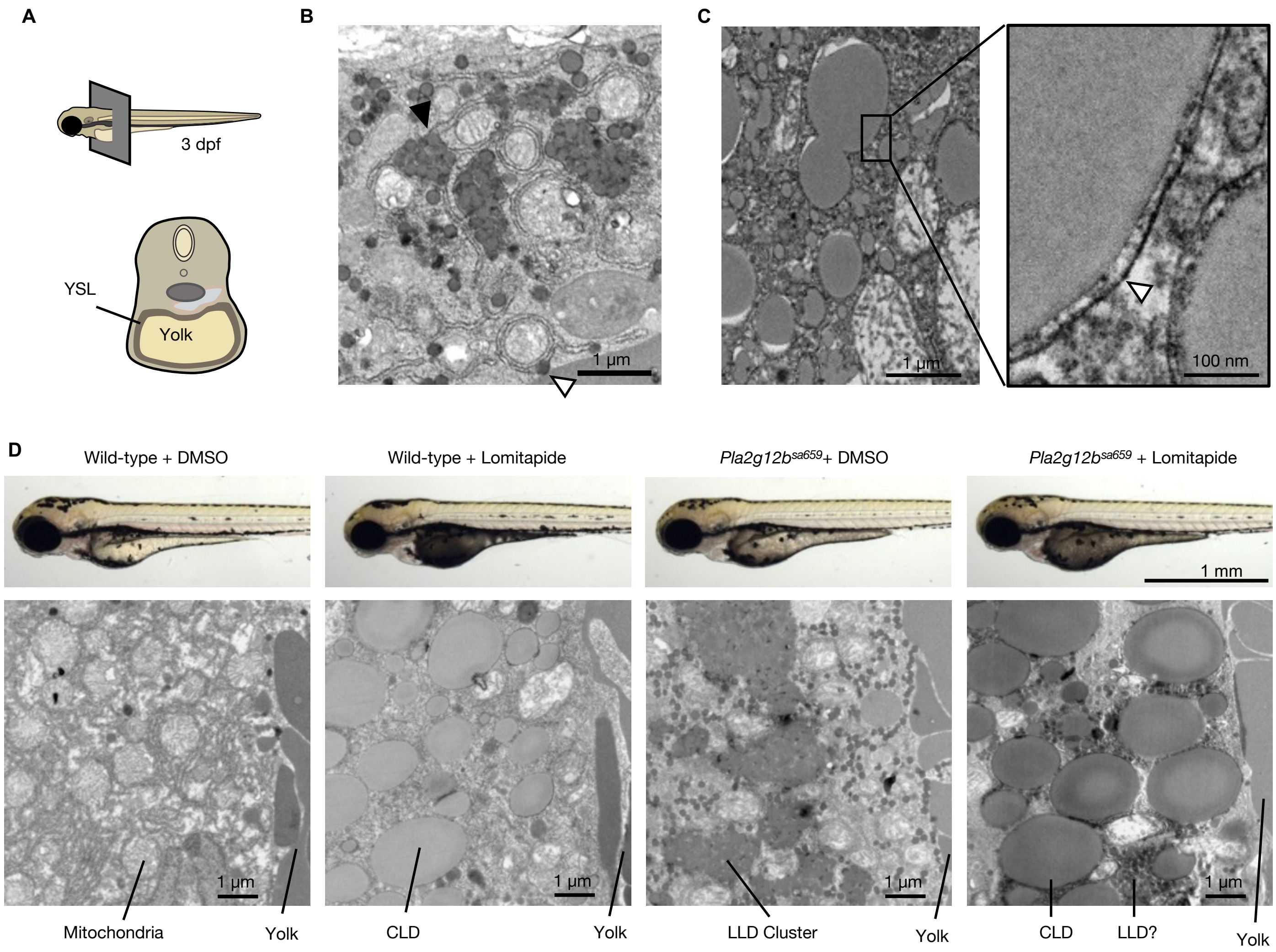

Supplementary Fig. 2: Electron micrographs of zebrafish YSL. (A) Schematic of sectioning protocol and region of interest (YSL) in transverse sections. **(B)** Representative micrograph showing individual lumenal lipid droplets (white arrowhead) and clusters of particles (black arrowhead) distending the ER lumen. **(C)** High-magnification micrograph demonstrating that very large lipid droplets resembling CLDs are contained within a secondary endomembrane (white arrowhead) in *pla2g12b*^{sa659} mutants. **(D)** Brightfield images and electron micrographs of wild-type and *pla2g12b*^{sa659} mutant exposed to the MTP inhibitor Lomitapide. MTP inhibition induces yolk darkening and CLD accumulation irrespective in both genotypes, suggesting that MTP acts upstream of Pla2g12b in the lipoprotein assembly pathway. Images are representative of n≥3 biological replicates.

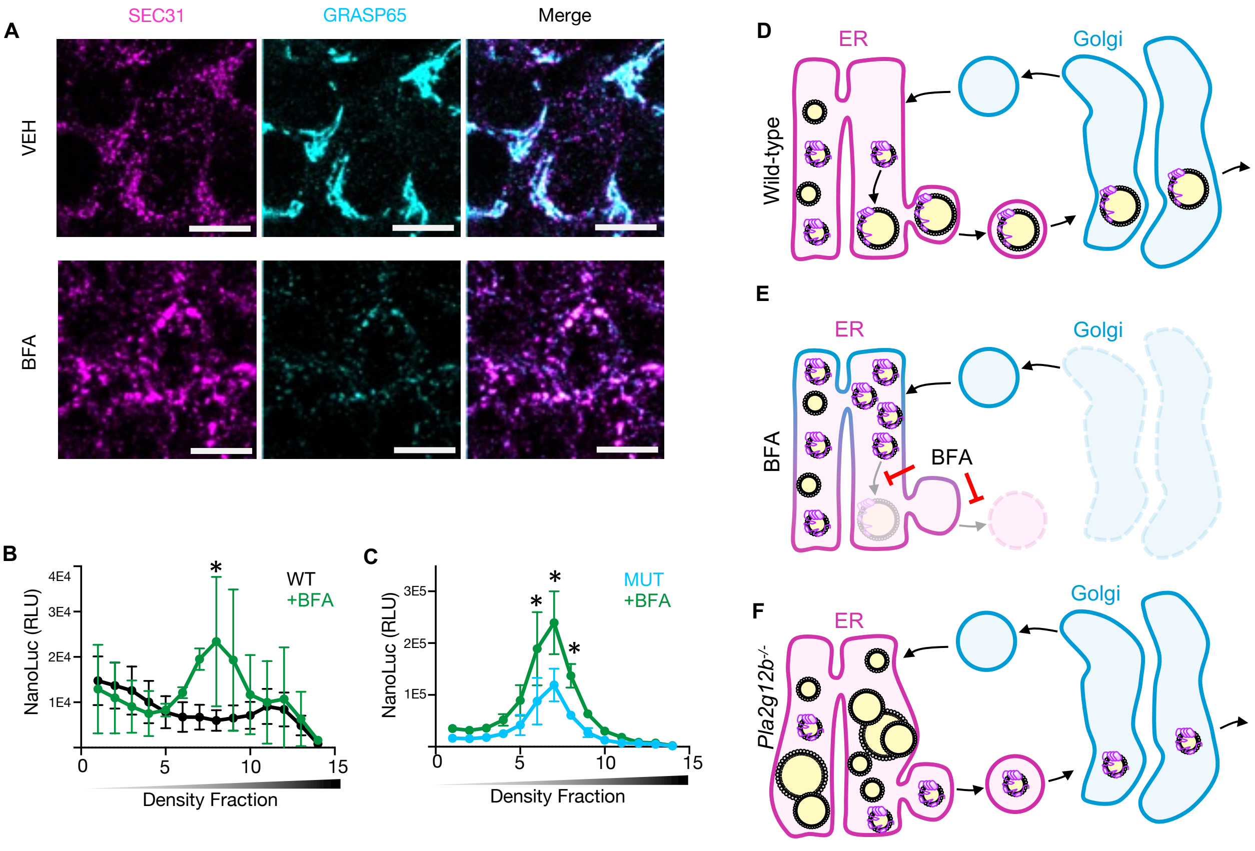

Supplementary Fig. 3: Mutations in *pla2g12b* disrupt TRL expansion but not secretion. **(A)** Representative confocal images of changes in ER-exit sites (marked by SEC31) and Golgi (marked by GRASP65) morphology in HepG2 cells treated with Brefeldin-A (BFA). BFA disrupts vesicular trafficking, blocking secretion and leading to dissolution of the Golgi and fusion with the ER. Scale bar = 10 µm. Images are representative of n≥3 biological replicates. **(B)** Density profiles of nascent TRLs from lumenal extracts of wild-type larvae (WT) exposed to BFA. BFA exposure led to the accumulation of dense TRLs within the microsomal lumen. Two-way ANOVA $p < 0.01$ for interaction at all stages, the asterisk denotes $p < 0.05$ by Šídák's multiple comparison test. **(C)** Density profiles of uminal TRLs from *pla2g12b* mutant larvae exposed to BFA demonstrate that BFA drives further accumulation of nascent TRLs without affecting the particle size distribution. Two-way ANOVA $p < 0.01$ for interaction at all stages, asterisk denotes $p < 0.05$ by Šídák's multiple comparison test. **(D)** Schematic representation of TRL expansion and secretion in wild-type larvae, and **(E)** BFA interferes with both TRL expansion and secretion. **(F)** By contrast, mutations in *pla2g12b* selectively disrupt TRL expansion but leave secretion intact. Elements of panels D, E, and F have been adapted from previous work (Thierer et al. 2019) under Creative Commons Attribution 4.0 International License.

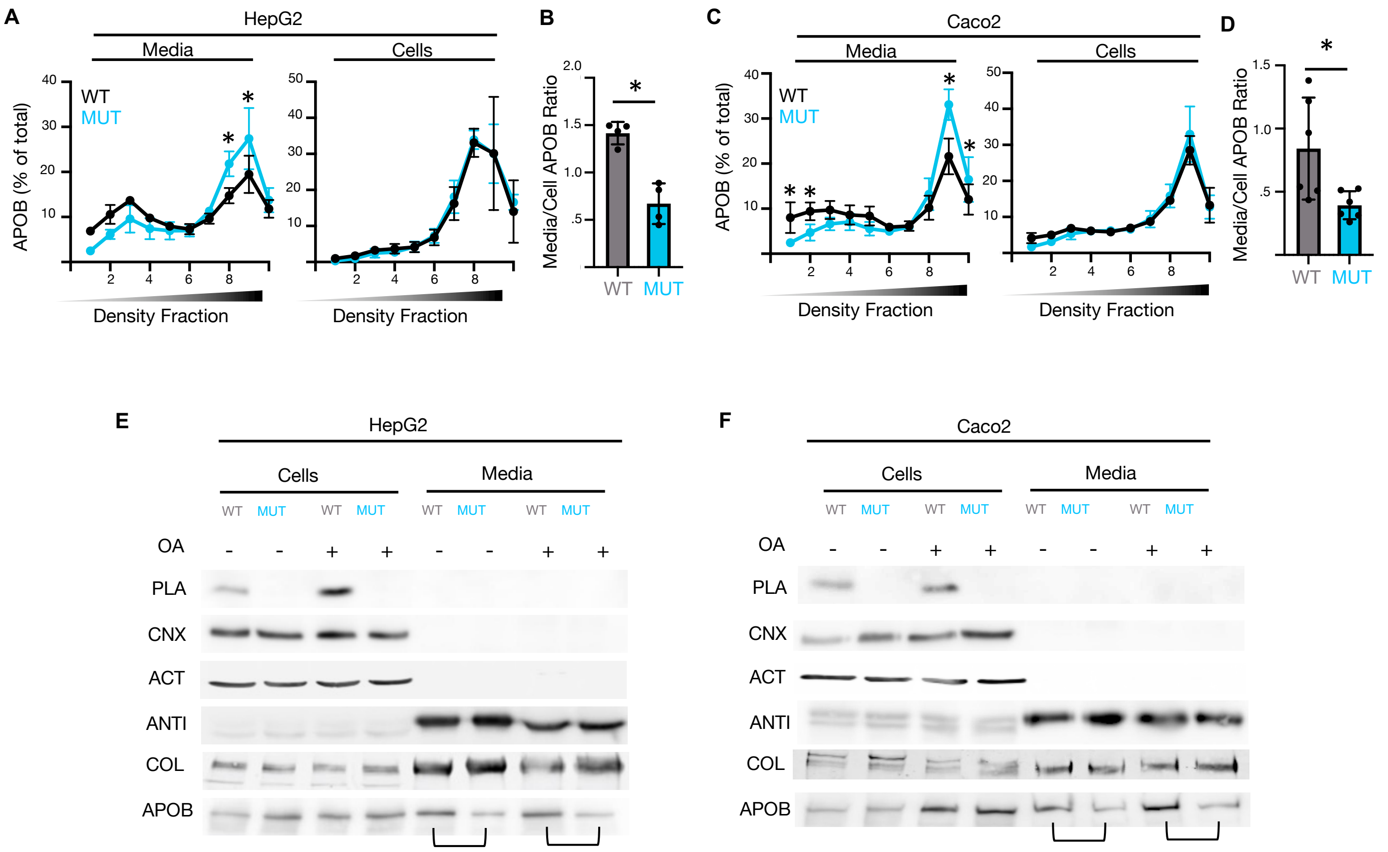

Supplementary Fig. 4: Lipoprotein expansion and secretion is disrupted in *PLA2G12B* mutant human cells. (A) Density profiles of APOB from the media of HepG2 cells normalized to total quantity of APOB secreted to highlight differences in density distribution. Mutant cells exhibit significant enrichment of dense TRLs in the media, but no significant changes are apparent in cell extracts. Data are mean ± s.d. of n ≥ 4 biological replicates. Two-way ANOVA $p < 0.0001$ for interaction in media samples only, the asterisk denotes $p < 0.05$ by Šídák's multiple comparison test. (**B)** Quantification of the media/intracellular APOB ratio shows significantly higher APOB secretion in WT cells. Unpaired two-tailed t-test, p = 0.0009. (**C)** Mutant Caco2 cells exhibit similar trends as HepG2 cells, with enrichment of dense TRLs and depletion of buoyant TRLs in the media. Two-way ANOVA $p < 0.0001$ for interaction in media samples only, the asterisk denotes $p < 0.05$ by Šídák's multiple comparison test. **(D)** Caco2 cells also exhibit reduced APOB secretion as measured by the ratio of APOB in cellular and media extracts. Unpaired two-tailed t-test, p = 0.0254. **(E)** Western blots monitoring impact of *PLA2G12B* genotype on secretion of various proteins from HepG2 and **(F)** Caco2 cells. PLA2G12B (PLA), CALNEXIN (CNX), and ACTIN (ACT) are not secreted. ANTI-TRYPSIN (ANTI) and COLLAGEN-XII (COL) are secreted normally irrespective of genotype, but the reduced APOB secretion in *PLA2G12B* mutants quantified in panels B and D above is also evident in Western blots (black brackets).

A

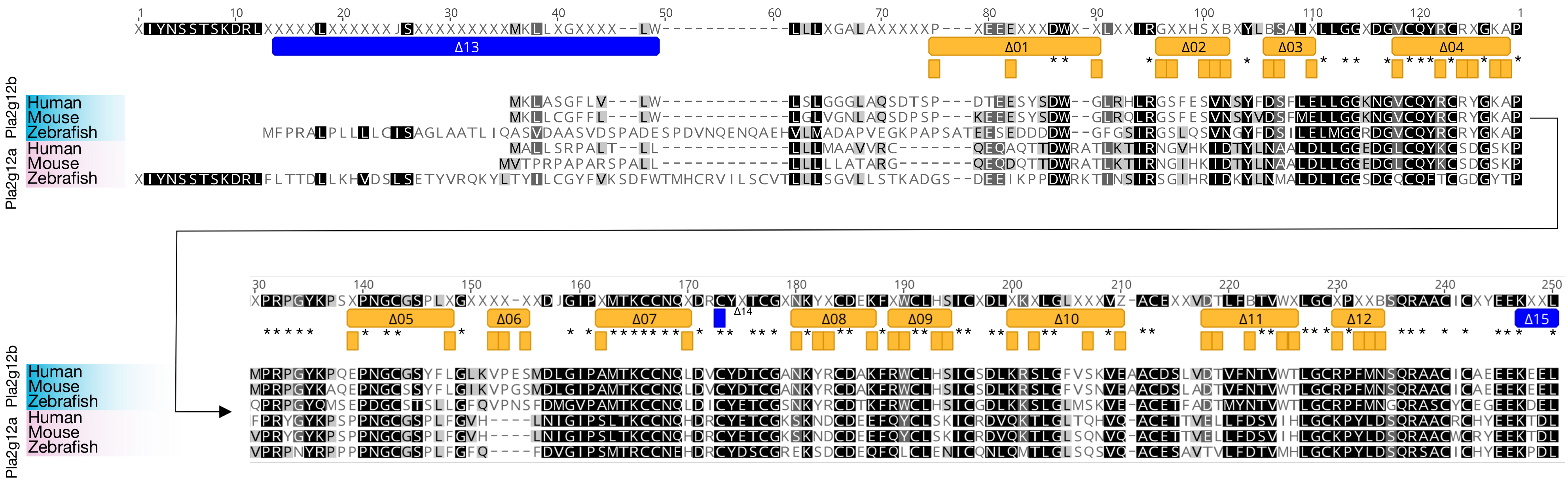

B

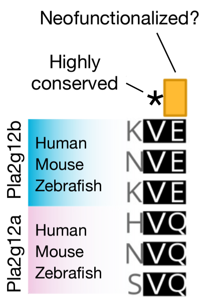

C

Allele	Substitutions / deletions
Δ01	P61G, E65I, G72T
Δ02	G78S, S79G, S82R, V83I, N84D
Δ03	D88N, S89M, E92D
Δ04	V100Q, R104T, R106G, Y107D, K109Y, A110T
Δ05	E121P, L130F
Δ06	V134X, P135X, S136X
Δ07	A143S, L151H
Δ08	N161E, Y163S, R164D, K168Q
Δ09	R170Q, W171L, H174E, S175N
Δ10	K181Q, S183T, S188Q, E191Q
Δ11	D198T, T199V, N202D, W205M, T206H
Δ12	R210K, F212Y, M213L, N214D
Δ13	No SP
Δ14	C→Y homologous to ENU allele
Δ15	No KDEL

Supplementary Fig. 5: Design and functional assessment of *pla2g12b* alleles. (A) Amino acid alignment of *Pla2g12b* and *Pla2g12a* homologs from zebrafish, mouse, and human. Amino acids are shaded by similarity, with darker shading indicating higher similarity at that position. **(B)** Legend for labeling scheme in panel a, illustrating that positions that are perfectly conserved (identical) between all proteins are marked with an asterisk. These positions are considered unlikely to be responsible for neofunctionalization of *pla2g12b*. Positions that are conserved within orthologs but differ between paralogs are denoted with an orange rectangle. These positions are considered putatively neofunctionalized, as the change is highly conserved in Pla2g12b but absent in Pla2g12a. **(C)** Tabular representation of pla2g12b alleles. Numbering is based on the position within the zebrafish Pla2g12b amino acid sequence.

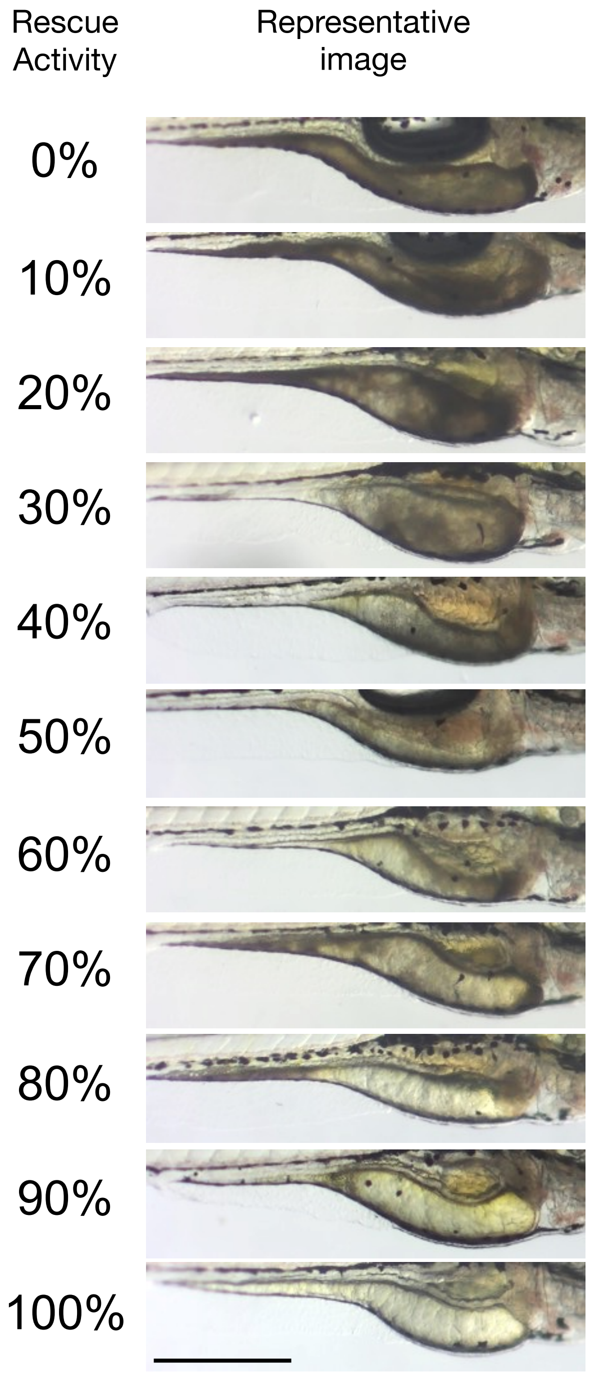

Supplementary Fig. 6: Representative scores for yolk rescue assay. Selected images from rescue injections and their corresponding scores, whereby the rescue activity was scored as the approximate percentage of the yolk surface area displaying the translucent appearance typical of wild-type larvae. Scale bar = 500 µm.

Supplementary Figure 7:

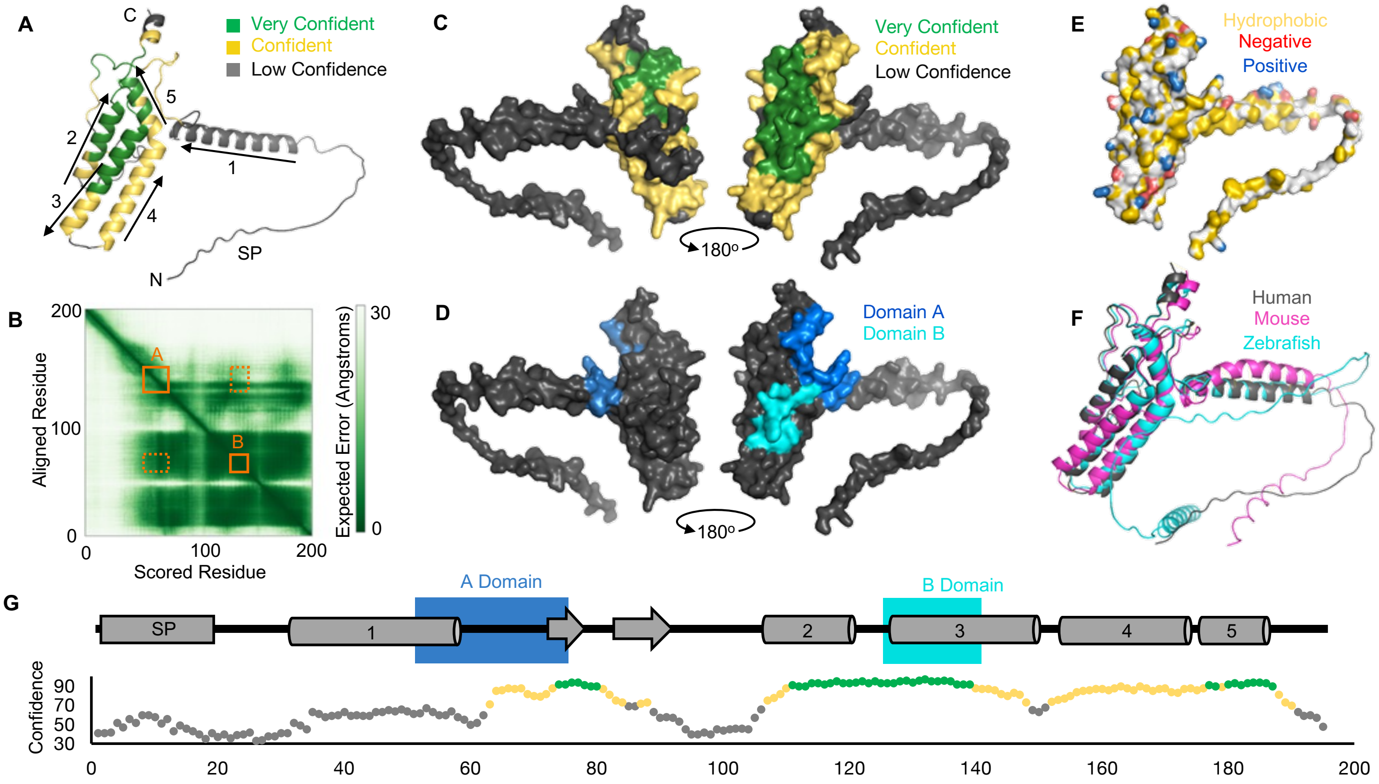

Supplementary Fig. 7: Predicted structure of Pla2g12b. (A) Predicted Structure of Pla2g12b generated by AlphaFold. Ribbon diagram is color-coded based on confidence of the 3D structure, and helix number and direction are indicated by labelled arrows. **(B)** Expected error plot with the essential domains A and B boxed in orange, demonstrating that the spatial relationship between these two domains has very low expected error (dashed orange boxes). (**C**) Space-filling model of Pla2g12b color coded by confidence. (**D**) Space filling model of Pla2g12b with essential domains A and B color coded in Blue and Cyan. **(E)** Space filling model of Pla2g12b color-coded by surface hydrophilicity. (**F**) Structural alignments of Pla2g12b structures from zebrafish, mouse, and human demonstrate significant overlap. (**G**) Linear view of predicted domain structure of Pla2g12b, with signal peptide (SP) denoted with a rectangle, helices shown as numbered cylinders, and beta sheets shown as arrows. Domains A and B are denoted with blue and cyan boxes, and the confidence of the predicted structure is shown below (confidence thresholds identical to panel a). Helix numbers and directions correspond to labels in panel a.

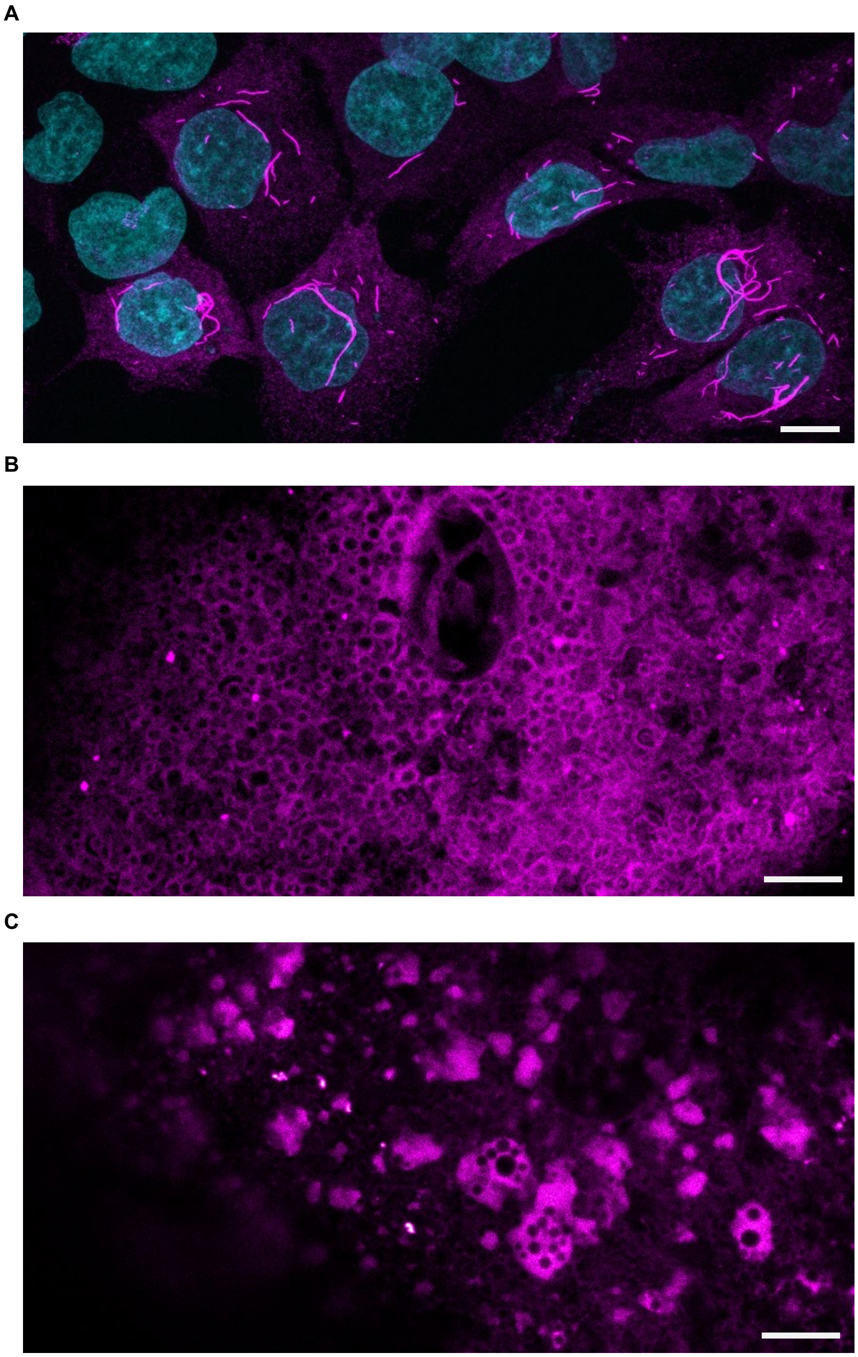

Supplementary Fig. 8: Confocal images of transgenic mScarlet-PLA2G12B fusion proteins. (A) Confocal image of mScarlet-PLA2G12B driven by a strong constitutive CMV promoter sequence in HepG2 cells. In addition to a faint reticular pattern throughout the cell, we also observe bright fibrous aggregates, which are likely to be an overexpression artifact. Scale bar = 10 µm. **(B)** Confocal micrograph of mScarlet-PLA2G12B driven by the YSL-specific *apo14* promoter in zebrafish larvae. Although no fibrous aggregates were detected, bright fluorescent puncta may reflect some level of protein aggregation in this context as well. The expression pattern is consistent with the network of tubules characteristic of the ER, although **(C)** fields in which the ER appears distended with lipid droplets as seen in Fig. 1G were also apparent. Scale bars = 10 µm. Images are representative of n≥3 biological replicates.

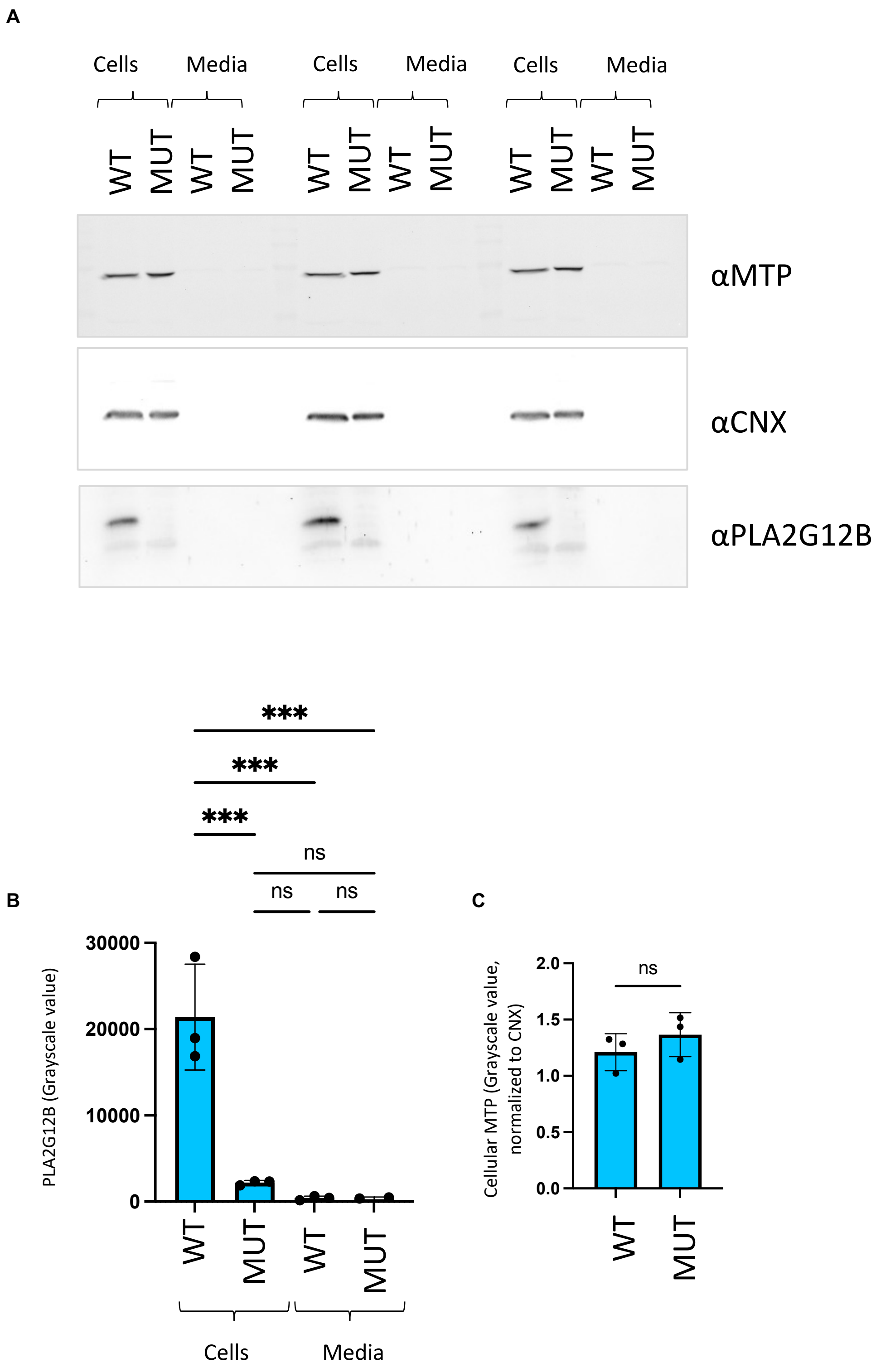

Supplementary Fig. 9: Quantification of cellular and secreted MTP and PLA2G12B. (A) Western blot on cellular and media protein extracts from WT and MUT cells performed in triplicate. **(B)** Quantification of PLA2G12B levels in the cells and media, demonstrating the PLA2G12B protein was virtually undetectable in mutant cells, and is not secreted appreciably into the cell culture medium. One-way ANOVA $p < 0.001$, the asterisk denotes $p < 0.0005$ by Tukey's multiple comparison test, $n = 3$. **(C)** Cellular MTP levels were not significantly different between wild-type and *PLA2G12B* mutant cell lines. Unpaired t-test, $p = 0.35$.

GeneSymbol	t-test	log2-fc	log2-abundance
dnajc3a	1.14976E-06	20.68243271	7.39472033
pla2g12b	7.65426E-05	20.65896745	22.26805782
apobb.1	0.030499149	3.399952677	17.77433903
p4hb	0.044879762	4.284491852	18.55466534
arcn1b	0.047649592	13.90972832	13.52799429
hspa5	0.070280042	2.332962656	19.73098657
nt5c2b	0.083238051	5.044439905	13.12123671
prdx4	0.086358992	4.918892077	15.41631984
arf4a	0.086790542	5.170837761	11.91208533
hsp90b1	0.088556229	3.266685512	17.8331628
hyou1	0.096853589	4.532544039	14.4857246
ube2d3	0.104045528	2.565240254	11.71611056
ube2d2l	0.104045528	2.565240254	11.71611056
ube2d2	0.104045528	2.565240254	11.71611056
rac1l	0.106494473	3.395118259	10.30299884
tpi1a	0.116116524	14.84076554	1.553053156
magt1	0.116118935	22.74993639	9.462224007
trappc12	0.116119797	19.84150742	6.553795036
atf1	0.116120058	19.37231354	6.08460116
crema	0.116120058	19.37231354	6.08460116
creb1b	0.11612012	19.20519092	5.917478539
tecrb	0.116127403	19.10360528	5.815892901
macrod1	0.116141882	17.66764519	4.379932812
LOC100537744	0.116145026	26.09451107	12.80679869
hsd17b12b	0.116147253	23.46483523	10.17712285
adssl	0.116147632	20.94026063	7.652548246
ttc7b	0.116154256	21.91623361	8.62852123
appl1	0.116240585	22.31687954	9.029167164
uggt1	0.116242012	24.10428774	10.81657536
vapal	0.116270527	24.3904316	11.10271923
slc25a6	0.116285288	25.99153982	12.70382744
gde1	0.116290107	18.44923433	5.161521954
tsta3	0.116308941	17.83347999	4.545767607
mrps17	0.116352273	12.60869491	13.61200399
mccc2	0.116404117	24.87578952	11.58807714
rsu1	0.116526578	22.13597713	8.848264751
pitpnb	0.116568154	20.14291055	6.855198175
adssl1	0.116620594	24.82319979	11.53548741
tuba1a	0.116647715	34.56355431	21.27584193
nudt9	0.116965206	24.82264761	11.53493523
eif4a1b	0.117173289	29.53318885	16.24547647
calb2b	0.117317102	25.90344313	12.61573075
h6pd	0.117512259	20.05276685	6.765054471
hdac10	0.117999452	19.17405472	5.886342341
zgc:136908	0.118170595	23.90478103	10.61706865
zgc:112271	0.120454202	1.338251142	14.10519099
hspa4a	0.121514621	22.98980329	9.702090912
rps26l	0.125324073	1.354704349	15.62628349
mttp	0.134528211	8.328166776	17.9536142

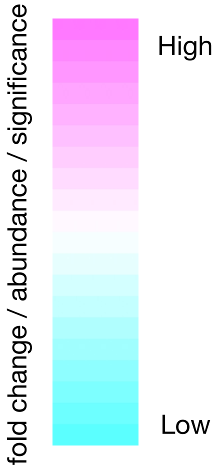

Supplementary Fig. 10: Co-immunoprecipitation and Mass-Spectrometry. Tabular representation of top 50 interacting partners meeting significance, enrichment, and abundance thresholds.

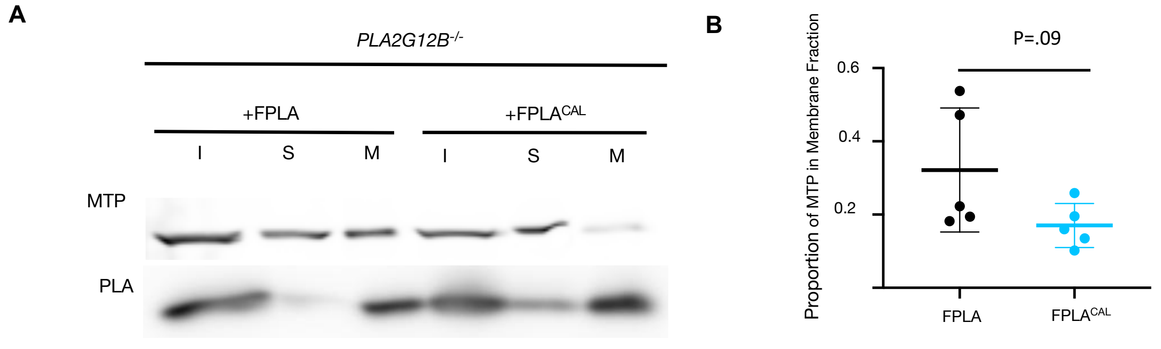

Supplementary Fig. 11: (A) Representative Western blot and **(B)** quantification showing membrane association of MTP in *PLA2G12B* mutant cells transfected with either 3xFLAG-tagged PLA2G12B (FPLA) or an allele with mutations in the Calcium-binding domain ($FPLA^{CAL}$). Data are from n = 5 biological replicates. Unpaired two-tailed t-test, $p < 0.05$.

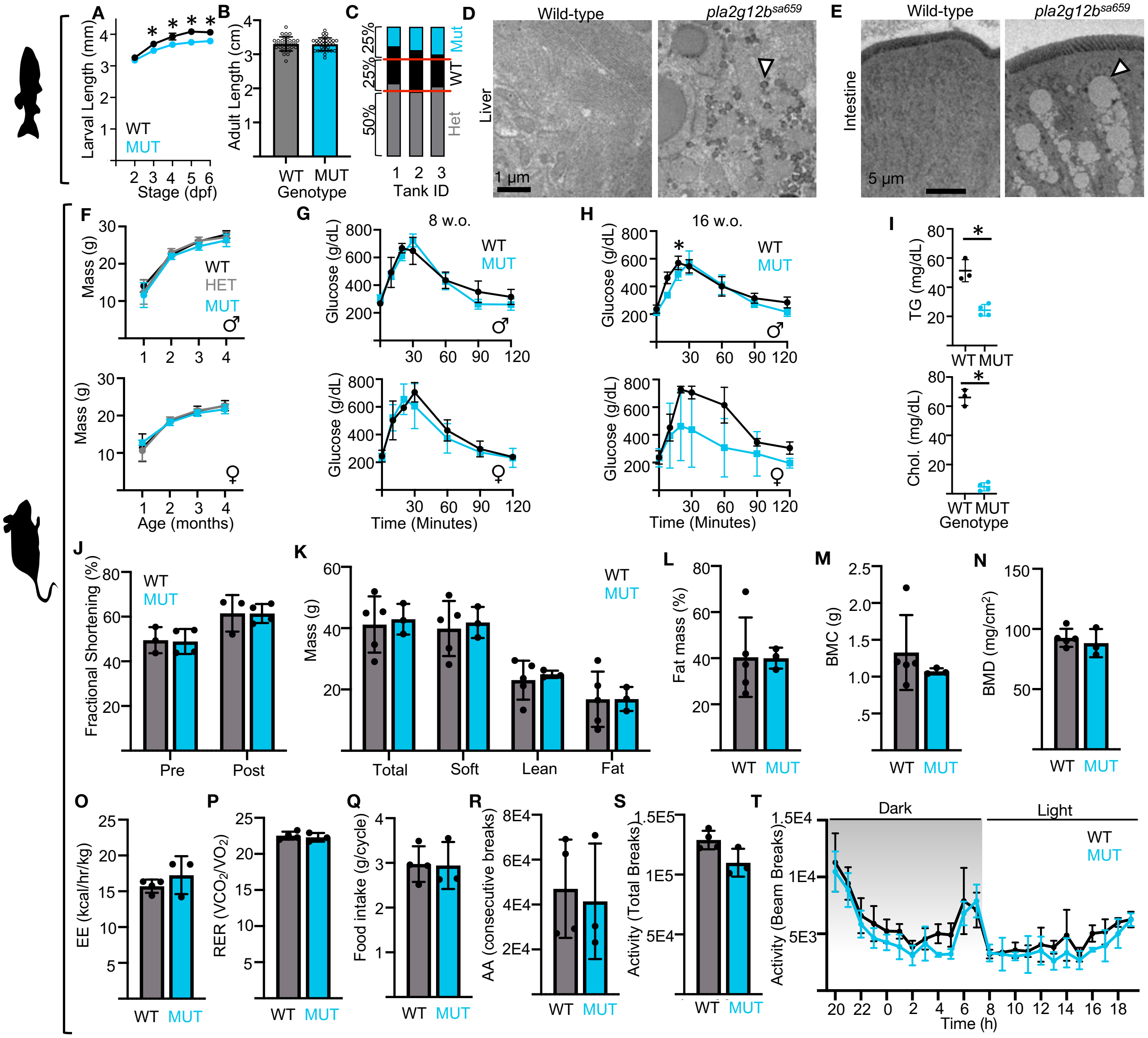

Supplementary Fig. 12: Mutations in *PLA2G12B* cause mild disruptions to systemic metabolism. (A) Body length is slightly reduced in *pla2g12b* mutant larvae. (**B)** Adult body size was not significantly different between *pla2g12b* mutant zebrafish and wild-type siblings. (**C)** Genotype ratios of surviving offspring were not significantly different from expected mendelian ratios across three independent clutches. (**D)** Electron micrographs of liver and (**E**) intestinal sections from adult zebrafish are indicative of excess lipid accumulation in lipoprotein-producing tissues following 24 hour fast. Images are representative of n≥3 biological replicates. (**F)** *Pla2g12b* genotype had no effect on body weight in male (upper panel) or female (lower panel) mice. (Two-way ANOVA p > 0.5 for both sexes). (**G)** Glucose tolerance was not significantly different in 8 week-old mice. (Two-way ANOVA p > 0.2 for both sexes). **(H)** Differences in glucose tolerance were detected in both sexes by 16 weeks of age. (Two-way ANOVA p < 0.005 for both sexes, the asterisk denotes p <. 005 by Šídák's multiple comparison). (**I)** Serum triglycerides (upper panel) and cholesterol (lower panel) were significantly reduced in *PLA2G12B* mutants. (Unpaired two-tailed t-test, p < 0.005). **(J)** Quantification of fractional shortening in mice subjected to a dobutamine stress test, showing there is no defect in cardiac function in *pla2g12b* mutants. (One-way ANOVA p > 0.9 for effect of genotype). (**K)** Body composition parameters from DEXA scan show no differences in total, soft, lean, or fat mass, nor (**L**) fat mass percentage, **(M)** bone mineral content, or **(N)** bone-mineral density. (Unpaired two-tailed t-test, p > 0.05) **(O)** Quantification of parameters from CLAMS assay showed no difference in energy expenditure, **(P)** respiratory exchange rate, **(Q)** food intake, or **(R)** ambulatory activity. **(S)** A slight reduction in total activity was detected in mutant males, but **(T)** was not restricted to a particular time of day. (Panels p-t: Not significant by unpaired t-test, p > 0.05. Panel u: Unpaired t-test p = 0.047, not significant after adjustment for multiple comparisons. Genotype had a significant effect on total activity by Two-way ANOVA, p < 0.0001, *post hoc* Šídák's test used for multiple comparisons was not significant at any individual time point).

A

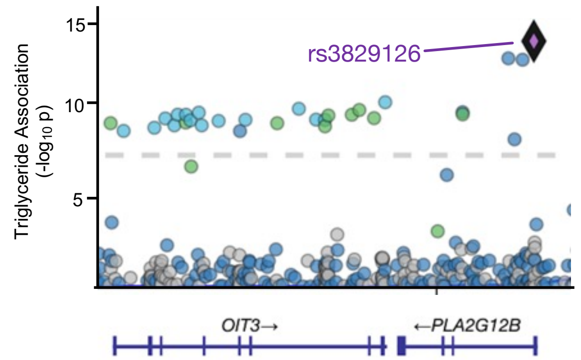

B

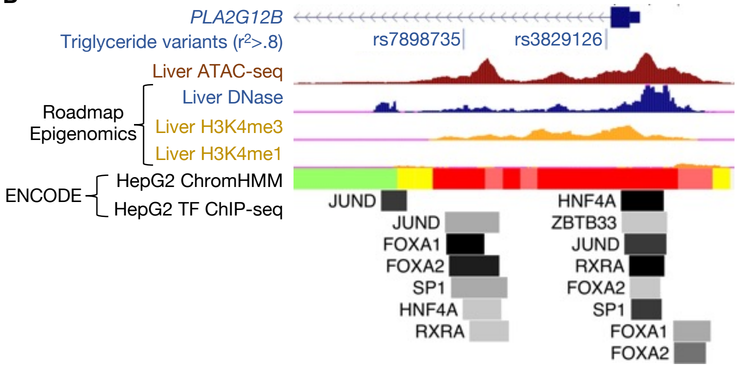

C

Phenotype	P-Value
Triglycerides	1.18e-26
LDL cholesterol	4.05e-23
HDL cholesterol	2.96e-11
Triglyceride levels in individuals without type 2 diabetes	6.96e-11
Alanine transaminase (ALT)	1.58e-10
AST-ALT ratio	1.64e-9
Alkaline phosphatase (ALP)	3.04e-9
Gamma-glutamyl transferase (GGT)	1.58e-7
Total cholesterol	1.93e-7
Plasma C-reactive protein	6.41e-6

D

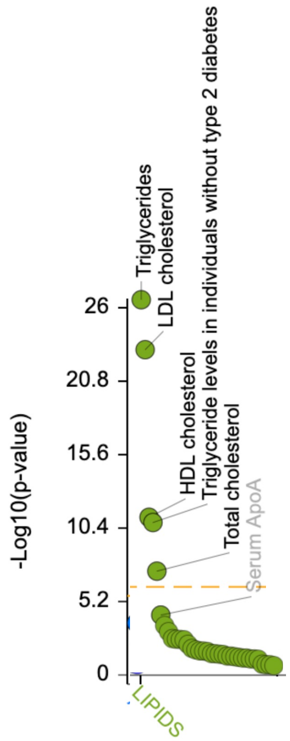

Supplementary Fig. 13: Human variants linked to *PLA2G12B* affect serum TG levels (A) DNA variants near the human *PLA2G12B* gene are significantly associated with plasma triglyceride levels**. (B)** Lead SNPs associated with triglyceride levels are located in regulatory regions in intron 1 of *PLA2G12B*. Red denotes active promoter, yellow denotes weak enhancer, and green denotes genic enhancer in the ChromHMM track. **(C)** Gene-level associations of traits from the type 2 diabetes knowledge portal and **(D)** graphical view of associations with lipid traits.

BD FACSDiva 8.0.2

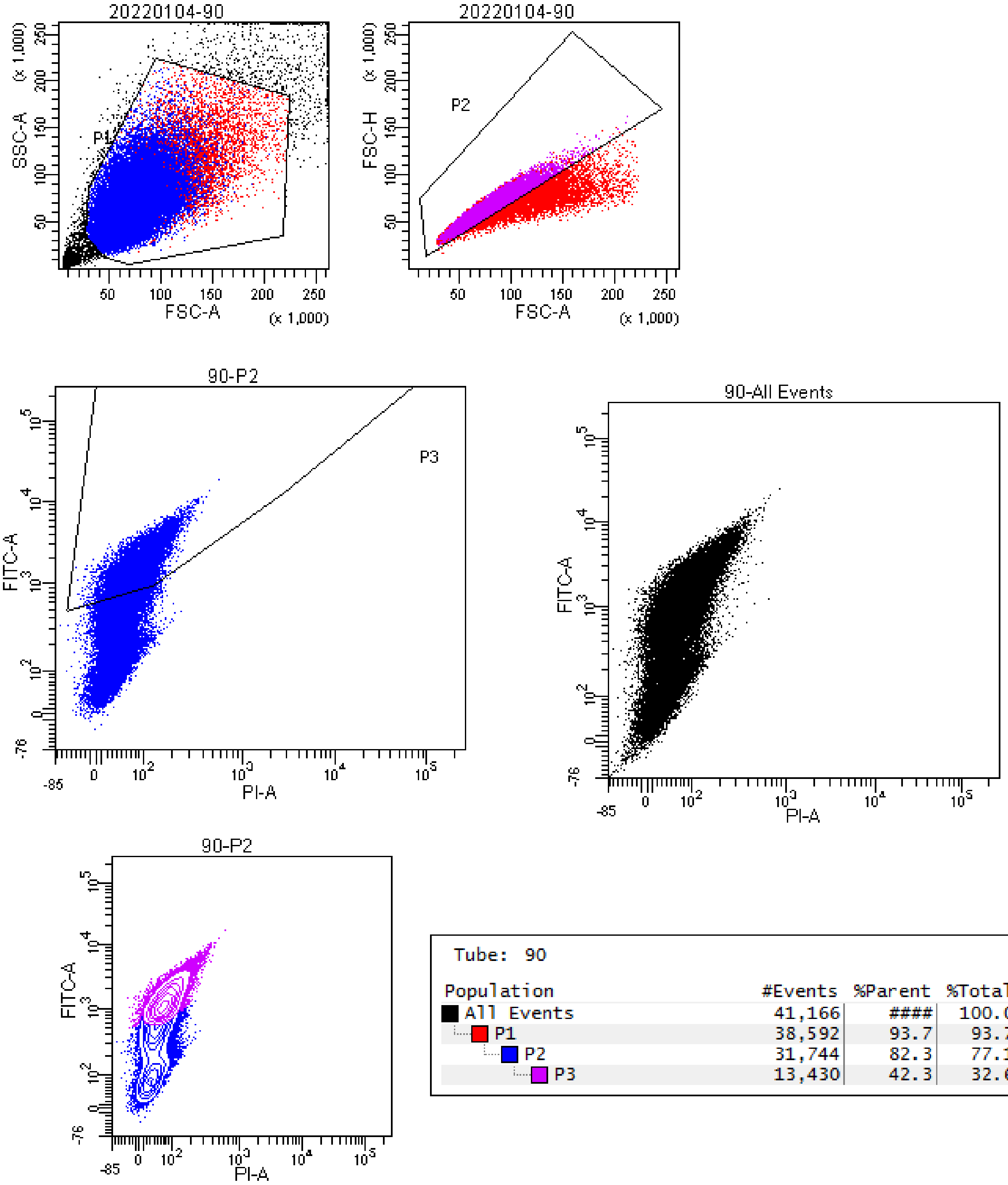

Tube: 90

Population	#Events	%Parent	%Total
All Events	41,166	####	100.0
P1	38,592	93.7	93.7
P2	31,744	82.3	77.1
P3	13,430	42.3	32.6

Supplementary Fig. 14: Report of gating strategies used to select GFP-positive HepG2 cells expressing the rescue alleles of *PLA2G12B*.

Supplementary Table 1: Plasmids used in this study

Promoter/ expression	Description	point mutations
CMV/Human	Human-WT	
CMV/Human	Mouse-WT	
CMV/Human	Zebrafish-WT	
CMV/Human	Human-FLAG-WT	
CMV/Human	Mouse-FLAG-WT	
CMV/Human	Zebrafish-FLAG-WT	
CMV/Human	Human-FLAG-delta-01	P61G, E65I, G72T
CMV/Human	Human-FLAG-delta-02	G78S, S79G, S82R, V83I, N84D
CMV/Human	Human-FLAG-delta-03	D88N, S89M, E92D
CMV/Human	Human-FLAG-delta-04	V100Q, R104T, R106G, Y107D, K109Y, A110T
CMV/Human	Human-FLAG-delta-05	E121P, L130F
CMV/Human	Human-FLAG-delta-06	V134X, P135X, S136X
CMV/Human	Human-FLAG-delta-07	A143S, L151H
CMV/Human	Human-FLAG-delta-08	N161E, Y163S, R164D, K168Q
CMV/Human	Human-FLAG-delta-09	R170Q, W171L, H174E, S175N
CMV/Human	Human-FLAG-delta-10	K181Q, S183T, S188Q, E191Q
CMV/Human	Human-FLAG-delta-11	D198T, T199V, N202D, W205M, T206H
CMV/Human	Human-FLAG-delta-12	R210K, F212Y, M213L, N214D
CMV/Human	Human-FLAG-delta-13	No SP
CMV/Human	Human-FLAG-delta-14	ENU C-->Y
CMV/Human	Human-FLAG-delta-15	No KDEL
CMV/Human	Human-FLAG-delta-16	CA
apo14/Zebrafish	Zebrafish-WT	
apo14/Zebrafish	Zebrafish-FLAG-WT	
apo14/Zebrafish	Zebrafish-mScarlet-WT	
apo14/Zebrafish	Zebrafish-FLAG-delta-01	P61G, E65I, G72T
apo14/Zebrafish	Zebrafish-FLAG-delta-02	G78S, S79G, S82R, V83I, N84D
apo14/Zebrafish	Zebrafish-FLAG-delta-03	D88N, S89M, E92D
apo14/Zebrafish	Zebrafish-FLAG-delta-04	V100Q, R104T, R106G, Y107D, K109Y, A110T
apo14/Zebrafish	Zebrafish-FLAG-delta-05	E121P, L130F
apo14/Zebrafish	Zebrafish-FLAG-delta-06	V134X, P135X, S136X
apo14/Zebrafish	Zebrafish-FLAG-delta-07	A143S, L151H
apo14/Zebrafish	Zebrafish-FLAG-delta-08	N161E, Y163S, R164D, K168Q
apo14/Zebrafish	Zebrafish-FLAG-delta-09	R170Q, W171L, H174E, S175N
apo14/Zebrafish	Zebrafish-FLAG-delta-10	K181Q, S183T, S188Q, E191Q
apo14/Zebrafish	Zebrafish-FLAG-delta-11	D198T, T199V, N202D, W205M, T206H
apo14/Zebrafish	Zebrafish-FLAG-delta-12	R210K, F212Y, M213L, N214D
apo14/Zebrafish	Zebrafish-FLAG-delta-13	No SP
apo14/Zebrafish	Zebrafish-FLAG-delta-14	ENU C-->Y
apo14/Zebrafish	Zebrafish-FLAG-delta-15	No KDEL
apo14/Zebrafish	Zebrafish-mscarlet-delta-16	CA
CMV/Human	Human-mScarlet-WT	
TetON/Human	Human-mScarlet-WT	
