# Supplementary material for: Pla2g12b drives expansion of triglyceride-rich lipoproteins
Source: Nat Commun. 2024 Mar 7;15:2095. doi: 10.1038/s41467-024-46102-4 (PMC10920679; doi:10.1038/s41467-024-46102-4)
Supplement: Supplementary file 3 — Reporting Summary [file 41467_2024_46102_MOESM3_ESM.pdf]

nature portfolio

Corresponding author(s): Steven A. Farber

Last updated by author(s): 2024/01/08

Reporting Summary

Nature Portfolio wishes to improve the reproducibility of the work that we publish. This form provides structure for consistency and transparency in reporting. For further information on Nature Portfolio policies, see our Editorial Policies and the Editorial Policy Checklist.

Statistics

For all statistical analyses, confirm that the following items are present in the figure legend, table legend, main text, or Methods section.

n/a	Confirmed	
☐	☒	The exact sample size (*n*) for each experimental group/condition, given as a discrete number and unit of measurement
☐	☒	A statement on whether measurements were taken from distinct samples or whether the same sample was measured repeatedly
☐	☒	The statistical test(s) used AND whether they are one- or two-sided<br>*Only common tests should be described solely by name; describe more complex techniques in the Methods section.*
☐	☒	A description of all covariates tested
☐	☒	A description of any assumptions or corrections, such as tests of normality and adjustment for multiple comparisons
☐	☒	A full description of the statistical parameters including central tendency (e.g. means) or other basic estimates (e.g. regression coefficient) AND variation (e.g. standard deviation) or associated estimates of uncertainty (e.g. confidence intervals)
☐	☒	For null hypothesis testing, the test statistic (e.g. *F*, *t*, *r*) with confidence intervals, effect sizes, degrees of freedom and *P* value noted<br>*Give P values as exact values whenever suitable.*
☒	☐	For Bayesian analysis, information on the choice of priors and Markov chain Monte Carlo settings
☒	☐	For hierarchical and complex designs, identification of the appropriate level for tests and full reporting of outcomes
☐	☒	Estimates of effect sizes (e.g. Cohen's *d*, Pearson's *r*), indicating how they were calculated

Our web collection on statistics for biologists contains articles on many of the points above.

Software and code

Policy information about availability of computer code

Data collection	FACS Diva (V8.0.2) software was used for data collection
Data analysis	Prism software (Version 9.3.1), RStudio (Version 1.4.1717), and Geneious (Geneious Prime 2022.2.1) were used.

For manuscripts utilizing custom algorithms or software that are central to the research but not yet described in published literature, software must be made available to editors and reviewers. We strongly encourage code deposition in a community repository (e.g. GitHub). See the Nature Portfolio guidelines for submitting code & software for further information.

Data

Policy information about availability of data

All manuscripts must include a data availability statement. This statement should provide the following information, where applicable:

- Accession codes, unique identifiers, or web links for publicly available datasets
- A description of any restrictions on data availability
- For clinical datasets or third party data, please ensure that the statement adheres to our policy

All data generated in this study have been deposited in the Harvard Dataverse database under accession code "Pla2g12b Drives Expansion of Triglyceride-Rich Lipoproteins” [https://doi.org/10.7910/DVN/6DFOSJ]. A subset of the data has also been included as a Source Data file associated with this paper.

Protein sequences were downloaded from Ensembl (ensemble.org) for pla2g12b from zebrafish (ENSDART00000128494.3, [https://useast.ensembl.org/

Danio_rerio/Transcript/Summary?db=core;g=ENSDARG00000015662;r=13:4671737-4683149;t=ENSDART00000128494]), mouse (ENSMUST00000009790.14, [https://useast.ensembl.org/Mus_musculus/Transcript/Summary?db=core;g=ENSMUSG00000009646;r=10:59239482-59257798;t=ENSMUST00000009790]), and human (ENST00000373032.4, [https://useast.ensembl.org/Homo_sapiens/Transcript/Summary?db=core;g=ENSG00000138308;r=10:72934762-72954806;t=ENST00000373032]), and pla2g12a from zebrafish (ENSDART00000103406.5, [https://useast.ensembl.org/Danio_rerio/Transcript/Summary?db=core;g=ENSDARG00000070454;r=1:12061284-12064799;t=ENSDART00000103406]), mouse (ENSMUST00000029629.15, [https://useast.ensembl.org/Mus_musculus/Transcript/Summary?db=core;g=ENSMUSG00000027999;r=3:129672255-129689474;t=ENSMUST00000029629]), and human (ENST00000243501.10, [https://useast.ensembl.org/Homo_sapiens/Transcript/Summary?db=core;g=ENSG00000123739;r=4:109709989-109730070;t=ENST00000243501]).

Research involving human participants, their data, or biological material

Policy information about studies with human participants or human data. See also policy information about sex, gender (identity/presentation), and sexual orientation and race, ethnicity and racism.

Reporting on sex and gender	*Use the terms sex (biological attribute) and gender (shaped by social and cultural circumstances) carefully in order to avoid confusing both terms. Indicate if findings apply to only one sex or gender; describe whether sex and gender were considered in study design; whether sex and/or gender was determined based on self-reporting or assigned and methods used. Provide in the source data disaggregated sex and gender data, where this information has been collected, and if consent has been obtained for sharing of individual-level data; provide overall numbers in this Reporting Summary. Please state if this information has not been collected. Report sex- and gender-based analyses where performed, justify reasons for lack of sex- and gender-based analysis.*
Reporting on race, ethnicity, or other socially relevant groupings	*Please specify the socially constructed or socially relevant categorization variable(s) used in your manuscript and explain why they were used. Please note that such variables should not be used as proxies for other socially constructed/relevant variables (for example, race or ethnicity should not be used as a proxy for socioeconomic status). Provide clear definitions of the relevant terms used, how they were provided (by the participants/respondents, the researchers, or third parties), and the method(s) used to classify people into the different categories (e.g. self-report, census or administrative data, social media data, etc.) Please provide details about how you controlled for confounding variables in your analyses.*
Population characteristics	*Describe the covariate-relevant population characteristics of the human research participants (e.g. age, genotypic information, past and current diagnosis and treatment categories). If you filled out the behavioural & social sciences study design questions and have nothing to add here, write "See above."*
Recruitment	*Describe how participants were recruited. Outline any potential self-selection bias or other biases that may be present and how these are likely to impact results.*
Ethics oversight	*Identify the organization(s) that approved the study protocol.*

Note that full information on the approval of the study protocol must also be provided in the manuscript.

Field-specific reporting

Please select the one below that is the best fit for your research. If you are not sure, read the appropriate sections before making your selection.

☒ Life sciences ☐ Behavioural & social sciences ☐ Ecological, evolutionary & environmental sciences

For a reference copy of the document with all sections, see nature.com/documents/nr-reporting-summary-flat.pdf

Life sciences study design

All studies must disclose on these points even when the disclosure is negative.

Sample size	As preexisting information on expected effect size and assay variability was not available, precise a priori calculation of sample sizes was not possible. Target sample sizes were therefore scaled to optimize the trade-off between experimental sensitivity and number of animal subjects used.
Data exclusions	Data were not excluded from analyses.
Replication	All experiments were replicated at least twice, with a replicate constituting unique biological material collected or analyzed on a different day, to verify reproducibility.
Randomization	Randomization was not relevant to our study design. All samples were either derived from an identical / clonal source (in the case of enzyme extracts and cultured cells), or gender-matched siblings were divided into equal groups. Therefore randomization of a mixed population was never necessary.
Blinding	Blinding was not relevant to all but one experiment because quantitative outputs eliminated the opportunity for experimenter bias. However, identifiers were removed prior to scoring rescue activity in zebrafish (Fig. 2C), effectively blinding researchers to group number. This was the only dataset in the paper for which image scoring was performed, and thus the only instance where blinding was necessary.

Reporting for specific materials, systems and methods

We require information from authors about some types of materials, experimental systems and methods used in many studies. Here, indicate whether each material, system or method listed is relevant to your study. If you are not sure if a list item applies to your research, read the appropriate section before selecting a response.

Materials & experimental systems

n/a	Involved in the study	
	x	Antibodies
	x	Eukaryotic cell lines
x		Palaeontology and archaeology
	x	Animals and other organisms
x		Clinical data
x		Dual use research of concern
x		Plants

Methods

n/a	Involved in the study	
x		ChIP-seq
	x	Flow cytometry
x		MRI-based neuroimaging

Antibodies

Antibodies used	The antibodies used for western blotting are as follows: rabbit anti-Antitrypsin Ab-1 (Neomarkers #RB-367-A, RRID:AB_59584, 1:2000 Dilution), Goat anti-Apolipoprotein B (TEBU-BIO #600-101-111, RRID:AB_2056958, 1:5000 Dilution), Rabbit anti-Calnexin (Abcam #ab22595, RRID:AB_2069006, 1:3000 Dilution), Rabbit anti-collagen XII (Santa Cruz #sc-68862, RRID:AB_2081581, 1:1000 Dilution), mouse anti-MTP C-1 (Santacruz #sc-515742, 1:1000 Dilution), Mouse anti-PDI C-2 (Santacruz #sc-74551, 1:1000 Dilution), Rabbit anti-PLA2G12B (Novus Biologicals #NBP231685, 1:1000 Dilution), Mouse anti-FLAG M2 (Sigma #F1804, RRID:AB_262044, 1:2000 Dilution), Mouse anti-beta actin clone AC-15 (Sigma #A1978, RRID:AB_476692, 1:2000 Dilution). The antibodies used for immunofluorescence were as follows: Goat anti-GRASP65 C-20 (Santa Cruz #sc-19481, RRID:AB_2232631, 1:500 Dilution), and Mouse anti-Sec31A (BD Biosciences #BD-612350, 1:500 Dilution).
Validation	Detailed information on antibody validation and related citations are available directly from the manufacturer's website using the product numbers listed above. Further, our generation of a PLA2G12B KO cell lines allowed us to further validate the specificity of the PLA2G12B antibody.

Eukaryotic cell lines

Policy information about cell lines and Sex and Gender in Research

Cell line source(s)	Commercially derived HepG2 (ATCC HB 8065, male) and Caco2 (ATCC HTB 37, male)
Authentication	Cell lines were not authenticated.
Mycoplasma contamination	Cell lines were not tested for Mycoplasma contamination.
Commonly misidentified lines (See ICLAC register)	Commonly misidentified cell lines were not incorporated into this study.

Animals and other research organisms

Policy information about studies involving animals; ARRIVE guidelines recommended for reporting animal research, and Sex and Gender in Research

Laboratory animals	All zebrafish were bred into the wild-type AB background. All mice were derived from the C57BL/6J-Pla2g12bhlb218/J background obtained from jackson labs. A variety of embryonic / adult stages were used throughout the study, and precise age of each organsims is reported in the associated figure legends.
Wild animals	This study did not involve wild animals.
Reporting on sex	Sex cannot be determined for zebrafish in the larval stages, so it can be excluded as a biological variable for these studies. For adult animals, analyses were performed independently on males and females, and in cases where results were consistent between sexes pooled statistical analyses were performed.
Field-collected samples	This study did not include field collected samples.
Ethics oversight	All research complies with all ethical guidelines, and approved by the Carnegie Institution Animal Care and Use Committee (Protocol #156) and NYU Langone Institution Animal Care and Use Committee (Protocol #201900083).

Note that full information on the approval of the study protocol must also be provided in the manuscript.

Plants

Seed stocks: N/A

Novel plant genotypes: N/A

Authentication: N/A

Flow Cytometry

Plots

Confirm that:

- [x] The axis labels state the marker and fluorochrome used (e.g. CD4-FITC).
- [x] The axis scales are clearly visible. Include numbers along axes only for bottom left plot of group (a 'group' is an analysis of identical markers).
- [x] All plots are contour plots with outliers or pseudocolor plots.
- [x] A numerical value for number of cells or percentage (with statistics) is provided.

Methodology

Sample preparation: For generation of stable HepG2 cell lines for rescue/complementation assays, The cDNA encoding for wild-type and mutant pla2g12b alleles were subcloned into pL309-GFP Lentiviral transfer plasmid77. The transgene sequence is followed by an IRES and the coding sequence of GFP to allow selection of infected cells. Lentiviral particles were generated by co-transfecting 1µg of the transfer plasmid (L309-GFP) containing the pla2g12b allele of interest with the packaging plasmids pRSV–REV, pMDLg/pRRE and envelope plasmid VSV-G (1 µg in total), with 6µl of TransIT reagent (Mirus Bio. Cat. No.: 293) into 0.8×106 HEK−293T (ATCC, negative for mycoplasma) cells plated in a 60mm culture dish containing 3ml of complete media. The following day the cell culture media was changed with fresh media. Two and three days after, the cell media was collected and filtered through a 0.45µm membrane. 0.8×106 PLA2G12B-/- HepG2 cells were plated in 60mm dishes. The following day the media was changed for 2ml of fresh complete media, 1ml of the filtered viral particles and 8µg/ml of hexadimethrine bromide (polybrene) (Sigma-Aldrich. Cat. No.: 107689). The following day media was change with fresh media.

Instrument: BD FACSAria III from BD Biosciences

Software: FACS Diva 8.0.2

Cell population abundance: 29-52.8% of cells met selection criteria /gating strategies across a variety of plasmid transfections.

Gating strategy: Cells were initially sorted based on SSC-A relative to FSC-A, and subsequently by FSC-H relative to FSC-A. Finally, a Fluorescent FITC selection window was selected with A PI counterstain for cell viability.

- [x] Tick this box to confirm that a figure exemplifying the gating strategy is provided in the Supplementary Information.
